# Evaluation of *LOXL1* polymorphisms in eyes with exfoliation glaucoma in Japanese

**Published:** 2008-07-21

**Authors:** Nobuo Fuse, Akiko Miyazawa, Toru Nakazawa, MingGe Mengkegale, Takaaki Otomo, Kohji Nishida

**Affiliations:** Department of Ophthalmology, Tohoku University Graduate School of Medicine, Sendai, Japan

## Abstract

**Purpose:**

To investigate the lysyl oxidase-like 1 (*LOXL1*) gene for single nucleotide polymorphism (SNP) variations in Japanese patients with exfoliation syndrome (XFS) and exfoliation glaucoma (XFG) and to examine the phenotypes of the patients with these variations.

**Methods:**

Fifty-six unrelated Japanese patients with XFS, including 36 patients with XFG, were studied. Genomic DNA was extracted from the leukocytes of peripheral blood, and three SNPs (rs1048661; p.Arg141Leu, rs3825942; p.Gly153Asp, and rs2165241) were identified. These SNPs were amplified by polymerase chain reaction (PCR), directly sequenced, and genotyped.

**Results:**

Two nonsynonymous variants in exon 1 of *LOXL1,* rs1048661 and rs3825942, were found to be strongly associated with XFS including XFG. The frequency of the T allele (0.964) in rs1048661 in eyes with XFS was much higher in controls (0.507) with a p value of 7.7x10^−18^. The odds ratio for the T allele in rs1048661 was 26.0 (95% confidence interval, 18.3–37.1). In the haplotype analysis, T-G was overrepresented in XFS subjects (p=7.7x10^−18^), showing a highly significant difference in frequency between primary open-angle glaucoma (POAG) and the control group (p=0.07), but the G-G and G-A haplotypes were less represented in XFS subjects (p=1.1x10^−11^ and p=1.0x10^−4^, respectively). However, an earlier study reported the strongest associated SNP with XFS and XFG, rs2165241, showed no association.

**Conclusions:**

SNPs of *LOXL1* (rs1048661; Arg141Leu and rs3825942; Gly153Asp) are highly associated with XFS in the Japanese population. However, unidentified genetic or environmental factors independent of *LOXL1* will most likely influence the phenotypic expression of the syndrome.

## Introduction

Exfoliation syndrome (XFS; OMIM 177650) is a generalized disorder of the extracellular matrix characterized clinically by the pathological accumulation of abnormal fibrillar material in the anterior segment of the eye predisposing the eye to glaucomatous optic neuropathy [1-3]. XFS has also been associated with weakness of the lens zonule fibers, severe chronic open-angle glaucoma, cataract formation, and a spectrum of other serious spontaneous intraocular defects. There is also evidence that XFS is associated with cardiovascular and cerebrovascular morbidity [2,4,5].

The prevalence of XFS varies markedly among populations and is highest in the Scandinavian population and markedly lower in Anglo-Celtic Caucasians [6-9]. The prevalence increases with age and is highest between 70 and 80 years of age [7]. Forty percent of Icelandic individuals 80 years or older in the Reykjavik Eye Study [10] and 22% of the population 70 years or older in the Finnish population were found to have XFS [11]. In the Japanese, the prevalence of XFS is reported to be between 1.1% (XFS with and without glaucoma, and glaucoma suspected of those over 40 years) to 4.8% (XFS with and without glaucoma, and glaucoma suspected of those over 80 years), which is much lower than the prevalence in the Scandinavian population [12,13].

Thorleifsson et al. [14] established a strong association between single nucleotide polymorphisms (SNPs) in the lysyl oxidase–like 1 (*LOXL1*) gene and XFS in the Swedish and Icelandic populations using a genome-wide scan. They identified two nonsynonymous SNPs (rs1048661; Arg141Leu and rs3825942; Gly153Asp) in exon 1 and one intronic SNP (rs2165241) in *LOXL1*, which conferred an attributable risk of 99% to Icelandic and Swedish individuals [14]. This association was recently confirmed in United States [15-17], Australian [18], and South Indian populations [19]. In addition, the SNPs, rs1048661 and rs3825942, of *LOXL1* seem to be highly associated with XFS in the Japanese population [20].

*LOXL1* is a member of the lysyl oxidase family of proteins that catalyzes the oxidative deamination of lysine residues of tropoelastin [21]. Elastic fiber homeostasis requires lysyl oxidase-like 1 protein [22], and this family of proteins has important roles in elastogenesis. Thus, it is biologically reasonable that defects in *LOXL1* may cause features of XFS because of the aberrant production of elastin and accumulation of fibrillar material in the anterior segment of the eye.

The purpose of this study was to investigate the lysyl oxidase-like 1 (*LOXL1*) gene for SNP variations in Japanese patients with XFS including those with exfoliation glaucoma (XFG) and compare them to patients with primary open-angle glaucoma (POAG) as well as to examine the phenotypes of the patients with these variations.

## Methods

### Patients

Fifty-six unrelated Japanese patients with XFS (31 men and 25 women; mean age 74.75±6.15 years) including 36 (23 men and 13 women) patients with XFG were studied. All of the patients resided in the northern part of Japan and were examined at the Ophthalmic clinic of the Tohoku University Hospital (Sendai, Japan). The purpose and procedures were explained to all patients, and an informed consent was obtained from each of them. This study was approved by the Tohoku University Institutional Review Board, and the procedures conformed to the tenets of the Declaration of Helsinki. Routine ophthalmic examinations were performed on all patients.

The criterion used for classifying a patient as having XFS was an open anterior chamber angle with accumulation of abnormal fibrillar material in the anterior segment of the eye. Patients who were diagnosed with XFG met the criterion of XFS and also had 1) applanation intraocular pressure (IOP) greater than 22 mmHg in each eye, 2) glaucomatous cupping in each eye including a cup-to-disc ratio greater than 0.7, and 3) presence of visual field defects determined by Goldmann perimetry and/or Humphrey field analyzer consistent with the glaucomatous cupping in at least one eye. The mean IOP at diagnosis was 35.0±12.0 mmHg in the 36 patients with XFG and 17.9±6.7 mmHg in the 20 patients with XFS but without glaucoma. Control subjects (76 men and 62 women; mean age 68.0±7.7 years) had the following characteristics 1) no accumulation of abnormal fibrillar material in the anterior segment of the eye, 2) IOP less than 22 mmHg, 3) normal optic discs, and 4) no family history of glaucoma. The mean IOP at the initial examination was 14.3±3.4 mmHg in the138 control patients.

Genomic DNA was extracted from peripheral blood leukocytes and purified with the Qiagen QIAamp Blood Kit (Qiagen, Valencia, CA), and the three SNPs (rs1048661, rs3825942, and rs2165241) were amplified by polymerase chain reaction (PCR), directly sequenced, and genotyped. For PCR, two primer sets (for rs1048661 and rs3825942 in exon 1: 5'-CTC AGC GCT CCG AGA GTA G-3' and 5'-ACA CGA AAC CCT GGT CGT AG-3'; for rs2165241 in intron 1: 5'-ACC AGG TAC TTG CAG CAT CC-3' and 5'-TGC TTG CCT AAT CAC AGC AC-3') were used under standard PCR conditions. The amplifications were performed at 60° C annealing temperature for the two SNPs in exon 1 and 58° C annealing temperature for the two SNPs in intron 1. The PCR fragments were purified by ExoSAP-IT (USB, Cleveland, OH) and sequenced by the BigDye^TM^ Terminator Cycle Sequencing Ready Reaction Kit (Perkin-Elmer, Foster City, CA) on an automated DNA sequencer (ABI PRISM^TM^ 3100 Genetic Analyzer, Perkin-Elmer).

The allelic frequencies, genotypes, and haplotypes of the patients with the *LOXL1* SNPs were determined. The allelic and genotypic frequencies were compared with two-tailed p values using χ^2^ analyses.

### Statistical analysis

The χ^2^ test, depending on cell counts, was used to determine if the genotype frequency differences between the cases and controls were significant. Odds ratios (approximating to relative risk) were calculated as a measure of the association between the *LOXL1* genotype and the phenotype of XFS. For each odds ratio, the p values and 95% confidence intervals were calculated. The inferred haplotypes, quantified between all pairs of biallelic loci, were estimated using the SNPAlyze program version 4.0 (Dynacom, Yokohama, Japan). Additionally, a permutation test was performed to test the deviation of allelic frequencies of SNPs and haplotypes [23]. The significance of the association was determined by contingency table analysis using the χ^2^ test. The Hardy–Weinberg equilibrium was analyzed using gene frequencies obtained by simple gene counting and the χ^2^ test with Yates' correction for comparing observed and expected values.

## Results

The allelic frequencies, genotypes, and haplotypes of the three *LOXL1* SNPs (rs1048661, p.Arg141Leu; rs3825942, p.Gly153Asp; and rs2165241 in intron 1) were determined in the cohort of subjects from the northern region of Japan.

### Distribution of *LOXL1* variants in exfoliation syndrome patients and control subjects

The T allele of the rs1048661 SNP was detected at a significantly higher frequency in patients with XFG and XFS without glaucoma than in the control subjects (p=1.7x10^−12^ and p=1.5x10^−8^, respectively; Table 1). Similarly, all of the XFS subjects had the G allele of rs3825942 SNP. The G allele was also associated with XFG and XFS without glaucoma (p=5.2x10^−3^ and p=0.027, respectively).

**Table 1:** *LOXL1* allelic frequencies of rs1048661 and rs3825942 in Japanese patients with exfoliation syndrome and controls.

**SNP**	**p.R141L (rs1048661 G/T)**			**Odds ratio (95%CI)**	**p value**	**p.G153D (rs3825942 G/A)**		**Odds ratio (95% CI)**	**p value**
Allele	T	G				G	A		
Japanese population in this study									
XFS all (n=56)	0.964	0.036		26.0 (18.3–37.1)	7.7x10^−18^	1	0	-	4.1x10^−4^
XFG (n=36)	0.958	0.042		22.2 (15.9–30.9)	1.7x10^−12^	1	0	-	5.2x10^−3^
XFS no glaucoma (n=20)	0.975	0.025		37.9 (25.0–57.5)	1.5x10^−8^	1	0	-	0.027
POAG (n=62)	0.605	0.395		1.49 (1.25–1.78)	0.11	0.911	0.089	1.43 (1.07–1.91)	0.37
Control (n=138)	0.507	0.493				0.877	0.123		
Japanese population in the Western Japanese study*									
XFS all (n=59)	0.992	0.008		N/A	3.0x10^−19^	1	0	-	1.4x10^−5^
XFG (n=27)	1	0		N/A	1.1x10^−10^	1	0	-	3.0x10^−3^
XFS no glaucoma (n=32)	0.984	0.016		N/A	1.7x10^−11^	1	0	-	1.3x10^−3^
Control (n=189)	0.54	0.46				0.857	0.143		
Icelandic population**									
XFG (n=75)	0.173	0.827	2.56 (1.74–3.77)		1.8x10^−6^	0.987	0.013	13.23 (5.59–31.29)	4.1x10^−9^
XFS no glaucoma (n=55)	0.211	0.789	2.02 (1.32–3.09)		1.3x10^−3^	0.982	0.018	10.10 (4.02–25.36)	8.5x10^−7^
POAG (n=90)	0.289	0.711	1.32 (0.96–1.82)		0.085	0.872	0.128	1.25 (0.81–1.91)	0.32
Control (n=14,474)	0.349	0.651				0.847	0.153		

The single asterisk indicates the data were reported by Hayashi et al. [20], and the double asterisk denotes that the data were reported by Thorleifsson et al. [14]. The significance of the associatio was determined by a contingency table analysis using the χ^2^ test. CI=confidence interval; N/A=Not applicable in the original report.

The genotypic frequencies for each of the two *LOXL1* SNPs were compared between XFS patients and control subjects (Table 2). The frequency of the T/T variant in the rs1048661 SNP was significantly higher in XFS patients than in control subjects (94.6% versus 21.7%), but the frequency of the T/G variant was significantly lower in the XFS patients than control subjects (3.6% versus 58.0%; p=1.6x10^−19^). There was no statistical difference between patients with POAG and control subjects.

**Table 2:** Frequency of genotypes, p.R141L, p. G153D, and rs2165241, of *LOXL1* in patients with XFS, XFG, and control subjects.

**Genotype**	**XFS all (n=56)**	**XFG (n=36)**	**XFS no glaucoma (n=20)**	**POAG (n=62)**	**Control (n=138)**
*LOXL1* p.R141L (rs1048661) variant					
T/T	53 (94.6%)	34 (94.4%)	19 (95.0%)	23 (37.1%)	30 (21.7%)
T/G	2 (3.6%)	1 (2.8%)	1 (5.0%)	29 (46.8%)	80 (58.0%)
G/G	1 (1.8%)	1 (2.8%)	0	10 (16.1%)	28 (20.3%)
p value*	1.6x10^−19^	7.8x10^−15^	3.0x10^−10^	0.15	
*LOXL1* p.G153D (rs3825942) variant					
G/G	56 (100%)	36 (100%)	20 (100%)	51 (82.3%)	108 (78.3%)
G/A	0	0	0	11 (17.7%)	26 (18.8%)
A/A	0	0	0	0	4 (2.9%)
p value*	7.5x10^−4^	8.8x10^−3^	0.068	0.58	
*LOXL1*rs2165241 variant					
C/C	54(96,4%)	34(94.4%)	20(100%)	56(91.3%)	122(88.4%)
C/T	2(3.6%)	2(5.6%)	0	6(9.7%)	16(11.6%)
T/T	0	0	0	0	0
p value*	0.08	0.29	0.11	0.69	

The asterisk indicates that the significance of the association was determined by a contingency table analysis using the χ^2^ test.

All of the patients with XFS had the G/G variant of rs3825942, which was higher in the XFS patients than in the control subjects (100% versus 78.3%; p=7.5x10^−4^). There was no significant difference between POAG patients and control subjects.

Interestingly, there was no statistically significant difference between XFS and XFG patients in the allelic frequencies of rs1048661 (p>0.05), and all the genotypes for rs3825942 were G-G in both XFS and XFG patients (Table 1). The strongest association of the SNPs with XFS was for the T allele of rs2165241, as in the original report [14]. However, there was no statistical difference between XFS, XFG, and controls in the allelic frequency of rs2165241 (Table 2). All SNPs adhered to the Hardy–Weinberg expectations (p>0.05).

### Haplotype analysis of *LOXL1* in the Japanese population

The inferred haplotypes between all pairs of biallelic loci were estimated (Table 3). Two SNPs, rs1048661 and rs3825942, were significantly associated with XFS (p<0.05). The haplotype-based associations were tested with a 1,000 iterated permutation test. Three major haplotypes, G-G, T-G, and G-A (each frequency greater than 5%), were found in the subjects, but the T-A haplotype was not found (Table 3). The T-G haplotype was overrepresented in all XFS patients, XFG patients, and XFS patients that did not have glaucoma (p=7.7x10^−18^, p=1.7x10^−12^, and p=1.5x10^−8^, respectively), showing a highly significant difference in frequency from the POAG and control groups, but the G-G and G-A haplotypes were less represented in all XFS patients, XFG patients, and XFS patients without glaucoma (Table3).

**Table 3:** *LOXL1* haplotypes in patients with exfoliation syndrome and in controls.

rs1048661 **-rs3825942Haplotype**	**XFS all (n=56)**	**p value**	**XFG (n=36)**	**p value**	**XFS no glaucoma (n=20)**	**p value**	**POAG (n=62)**	**p value**	**Control (n=138)**
G-G	0.0357	1.1x10^−11^	0.0417	4.1 x10^−8^	0.025	1.0 x10^−5^	0.3115	0.21	0.3768
T-G	0.9643	7.7x10^−18^	0.9583	1.7x10^−12^	0.975	1.5 x10^−8^	0.5984	0.07	0.5
G-A	0	1.0x10^−4^	0	1.7x10^−3^	0	0.02	0.0902	0.34	0.1232

The inferred haplotypes, quantified between all pairs of biallelic loci, were estimated using the SNPAlyze program version 4.0 (Dynacom, Yokohama, Japan).

### Phenotypes of exfoliation glaucoma patients in our population

The phenotypes of our 36 XFG patients were high IOP (35.0±12.0 mmHg), poor response to topical medication (number of eye drops; 2.4±0.4 bottles), aggressive course without treatment, and moderate response to filtration surgery. Among the XFS subjects, only one patient, a 76-year-old man with XFG, had a unique haplotype of G-G.

## Discussion

We confirmed the findings of Thorleifsson and colleagues [14] that two coding SNPs in *LOXL1* were strongly associated with XFS in our Japanese population. In recent studies, the frequencies of *LOXL1* SNP alleles, genotypes, and haplotypes detected in cohorts in Iowa and Australian were remarkably similar to those reported in Scandinavian populations [15,18]. The G allele of each of the *LOXL1* SNPs was highly associated with XFS, and the risk for disease in those populations was associated most strongly with the G-G haplotype.

However, the distribution of the rs1048661 alleles and haplotypes in patients were quite different between Scandinavian, Australian, American, and Japanese subjects. In the Northern Japanese in this study, the T allele of the rs1048661 SNP was dominant in controls, and more than 90% of XFS subjects had the T allele. These results reconfirmed the previous study in the Japanese population derived from the western part of Japan [20]. The frequency and position of the SNPs are different among various populations. So, it is difficult to apply the genome information of a polymorphism of Caucasians to that of East Asians. However, as for the Japanese population, the genetic background is homogeneous, and Table 1 showed the similarity between the Japanese population.

In general, Japanese patients with XFG associated with a *LOXL1* SNP show late-onset of glaucoma after 65 years of age. XFG would be a relatively uncommon age-related disease characterized by a generalized fibrillar degeneration of elastin-containing tissues. In our study, the age at onset was mainly older than 60 years of age (two case showed an onset of 55 and 58 years). The phenotypes of our XFG patients with the two variants were high IOP, poor response to topical medication, aggressive course without treatment, and probably moderate response to filtration surgery.

Recently, the most promising locus for diagnosing XFS was assigned to 18q12.1–21.33, and the susceptible loci were proposed to be 2q, 17p, and 19q in the Finnish population in an autosomal dominant mode of inheritance with incomplete penetrance [24]. In a microarray study, it was reported that the differentially expressed genes with a high level of reproducibility in patients with XFS were mainly related to extracellular matrix metabolism and cellular stress [25]. Hewitt et al. [18] described a remarkably similar *LOXL1* allelic architecture in the Blue Mountains Eye Study (BMES) where XFS is a relatively uncommon disease compared to that in Australia and to that of the Nordic population in which the XFS is extremely common. This strongly suggests that an as yet unidentified genetic or environmental factor independent of *LOXL1* strongly influences the phenotypic expression of the syndrome.

High risk *LOXL1* haplotypes account for a significant fraction of patients with XFS and thus may be useful in genetic screening tests. However, because many patients that carry *LOXL1* risk alleles do not have XFS, genetic testing for these alleles may be of limited usefulness at present. An unaffected control group with the highest risk diplotype (50%) would be expected to contain affected but not yet diagnosed individuals with XFS. Identification of XFS-associated SNPs of such magnitude that is clearly identifiable will allow early detection even before elevation of IOP at a later age or irreversible visual impairment due to damage of the optic nerve. More studies of the functions and genotype-phenotype correlation of *LOXL1* are required to determine the pathophysiology of XFS, and more studies such as meta-analysis with a greater number of subjects would be needed to confirm these genetic data.
